# Supplementary figures and images for: Research on state perception of scraper conveyor based on one-dimensional convolutional neural network
Source: PLoS One. 2024 Oct 18;19(10):e0312229. doi: 10.1371/journal.pone.0312229 (PMC11488722; doi:10.1371/journal.pone.0312229)

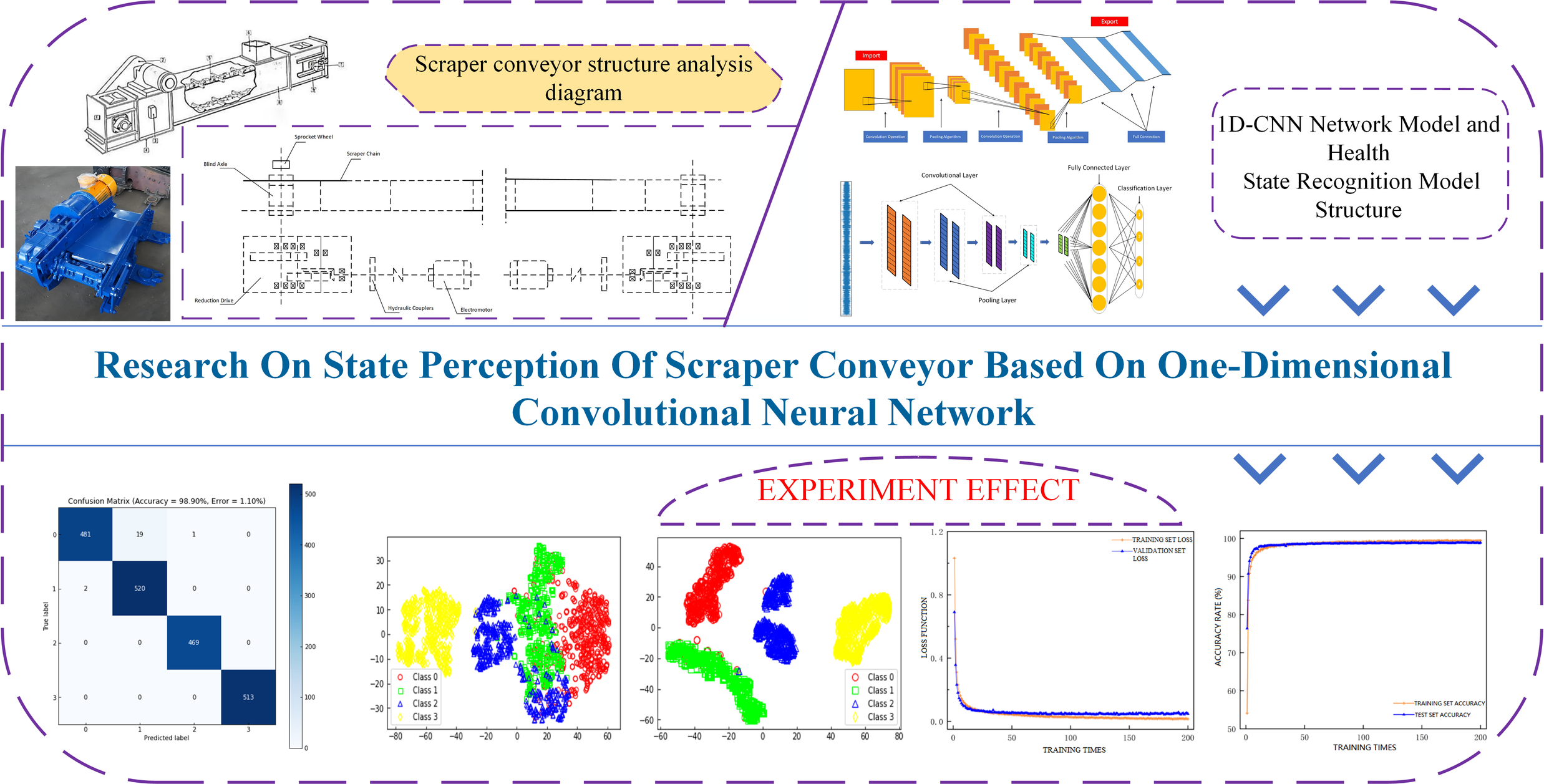

Supplement: S1 Graphical abstract — (TIF) [file pone.0312229.s002.tif]
