# Supplementary material for: Research on state perception of scraper conveyor based on one-dimensional convolutional neural network
Source: PLoS One. 2024 Oct 18;19(10):e0312229. doi: 10.1371/journal.pone.0312229 (PMC11488722; doi:10.1371/journal.pone.0312229)
Supplement: Attachment — Submitted filename: Rebuttal Letter.doc [file pone.0312229.s004.doc]

Dear Editor Gupta,

We are submitting our revised manuscript titled “Research on state perception of scraper conveyor based on one-dimensional convolutional neural network” (Manuscript ID: PONE-D-24-05986R1) for reconsideration at *PLOS ONE*. We sincerely appreciate the insightful comments and suggestions from you and the reviewers, which have greatly contributed to improving our manuscript. Below, we provide detailed responses to each comment and describe the revisions made.

**Comments to the Author**

1. If the authors have adequately addressed your comments raised in a previous round of review and you feel that this manuscript is now acceptable for publication, you may indicate that here to bypass the “Comments to the Author” section, enter your conflict of interest statement in the “Confidential to Editor” section, and submit your "Accept" recommendation.Reviewer #1: All comments have been addressedReviewer #2: All comments have been addressed

2. Is the manuscript technically sound, and do the data support the conclusions?The manuscript must describe a technically sound piece of scientific research with data that supports the conclusions. Experiments must have been conducted rigorously, with appropriate controls, replication, and sample sizes. The conclusions must be drawn appropriately based on the data presented.Reviewer #1: YesReviewer #2: Yes

3. Has the statistical analysis been performed appropriately and rigorously?Reviewer #1: N/AReviewer #2: No

Response: Thank you for your detailed review and valuable feedback on our paper. Regarding your concerns about the rigor of the statistical analysis, we would like to provide the following explanation and clarification:This study aims to propose a method based on a one-dimensional convolutional neural network (1DCNN) for assessing the health status of scraper conveyors. Compared to traditional methods that rely on expert knowledge or face difficulties in establishing degradation models, our innovation lies in the automatic extraction of features and health status recognition using the 1DCNN. This approach primarily focuses on evaluating the performance of the machine learning model, rather than traditional statistical inference or hypothesis testing, which is why extensive use of traditional statistical analysis was not incorporated in the study.In our research, the experiments were conducted using real monitoring signal data, covering various health conditions of scraper conveyors. By constructing a one-dimensional convolutional neural network, we extracted key features from the input signals and successfully mapped these features to the health status of the equipment using the model. The final experimental results demonstrated that the model achieved a recognition accuracy of 98.9%. To ensure the reliability of the experiments, we conducted multiple experiments across different datasets and rigorously evaluated the model using common machine learning performance metrics such as accuracy.We understand the importance of statistical analysis in certain types of research, especially in the field of data inference. However, as this study focuses on health status recognition through a deep learning model, traditional statistical analysis is not the main focus. Based on the experimental data and machine learning performance metrics, we believe that the proposed method is highly applicable and reliable in solving real-world problems.We hope that the above explanation addresses your concerns regarding statistical analysis. If you have any further questions on this topic or additional suggestions, please feel free to let us know, and we will do our best to supplement and improve the work.

4. Have the authors made all data underlying the findings in their manuscript fully available?

Reviewer #1: Yes

Reviewer #2: (No Response)

Is the manuscript presented in an intelligible fashion and written in standard English?Reviewer #1: YesReviewer #2: Yes

Review Comments to the AuthorReviewer #1:

Comment:Well-done you have incorporated all suggestions. Some Figures are not visible, kindly provide clear visible figures.

Response: Thank you for acknowledging the revisions we made. Regarding the issue you raised about some figures not being clear enough, we have reprocessed the relevant figures to enhance their resolution and visibility, ensuring they are clear on all reading devices and screens.

Reviewer#2:

1. Comment: The author describes the innovation of this article as "handling real-world noisy data". If the author's innovation mainly focuses on processing real-time noisy data, then the author's Introduction should summarize the literature in the field of processing real-time noisy data to reflect the author's innovation.

Response: Thank you for your valuable suggestions. The main innovation of this study lies in the precise identification of the health status of the scraper conveyor through the construction of a one-dimensional convolutional neural network (1DCNN), rather than focusing on noise processing. However, even though noise processing is not the core of this research, we have still implemented effective measures during the data processing stage to improve the model's adaptability to noisy data. Additionally, the Dropout layer applied in the 1DCNN model further enhances the model’s ability to handle noisy data. By randomly dropping certain neurons, Dropout prevents the model from over-relying on specific neurons, thereby improving its robustness when dealing with uncertain data, such as noisy data. Moreover, the convolutional filters of the 1DCNN can automatically extract local features from monitoring data, effectively distinguishing noise from meaningful health status features. This automated feature extraction capability not only improves the accuracy of the model’s identification but also reduces the impact of noise on health status assessment, providing solid technical support for the health management of scraper conveyors.The relevant references have been added as follows:Tan, P.S., Lim, K.M., Tan, C.H. et al. ComSense-CNN: acoustic event classification via 1D convolutional neural network with compressed sensing. SIViP 17, 735–741 (2023).Chen Kun, Wang Anzhi. A Review of Regularization Methods for Convolutional Neural Networks [J]. Computer Applications Research, 2024, 41(04): 961-969.Zhu Xiong, Song Wei, Guan Hongpeng, et al. Prediction of Stratum Pressure Curves Based on Convolutional Neural Networks with MC Dropout [C] // Petroleum Geophysical Professional Committee of the Chinese Petroleum Society. Proceedings of the Second Annual Academic Conference on Petroleum Geophysics in China (Volume II). China University of Petroleum (Beijing); 2024: 3.

2. Comment: The author cited the application of LSTM algorithm for fault diagnosis in the Introduction, and the author can use LSTM algorithm for comparison to highlight the author's innovation.

Response:

Thank you to the reviewer for your valuable suggestions. Regarding the application of the LSTM algorithm in fault diagnosis, as mentioned in the introduction, it indeed has advantages when dealing with time-series data with long-term dependencies. However, the 1DCNN model used in this study also has its own unique advantages, especially in practical applications for equipment health status evaluation.First, 1DCNN can efficiently extract local features when processing multi-channel, high-frequency monitoring data, and has lower computational complexity, making it suitable for real-time monitoring tasks. Compared to the application of LSTM in long-sequence data, 1DCNN achieves effective data dimensionality reduction through convolution and pooling operations, reducing model complexity and computation. In contrast, while LSTM excels at capturing long-term dependencies, its larger number of parameters, longer training time on large-scale industrial data, and tendency to overfit limit its application in scenarios requiring high real-time performance.The choice of 1DCNN as the monitoring model in this study is based on its superior performance in handling high-frequency, noisy industrial signals. 1DCNN not only quickly perceives the health status of equipment but also improves the model’s generalization capability and robustness against noise through the use of Dropout and pooling layers. Although LSTM performs well in certain scenarios, given the focus on high real-time performance and complex industrial settings in this study, 1DCNN is more appropriate. In future research, we will consider further comparing the performance of LSTM and 1DCNN to provide a more comprehensive performance analysis.The relevant references have been added as follows:(1) Mortezapour Shiri, Farhad & Perumal, Thinagaran & Mohamed, Raihani. (2023). A Comprehensive Overview and Comparative Analysis on Deep Learning Models: CNN, RNN, LSTM, GRU. 10.13140/RG.2.2.11938.81609.(2) Li Hanlin, Niu Shaozhang, Wang Maosen, et al. Real-time Detection Method for Equipment Abnormal Response Based on Feature Mining [J]. Artificial Intelligence, 2023, (04): 71-81. DOI: 10.16453/j.2096-5036.2023.04.008.(3) Sun Yan. A Brief Discussion on the Optimization of Convolutional Neural Networks [C] // Tianjin Electronics Society. Proceedings of the 37th China (Tianjin) 2023 IT, Network, Information Technology, Electronics, and Instrumentation Innovation Academic Conference. Tianjin Optoelectronic Communication Technology Co., Ltd.; 2023: 4.

We believe that the revised manuscript has been significantly improved and addresses all the concerns raised. We hope that the changes meet with your approval and that the manuscript is now suitable for publication in *PLOS ONE*.

Thank you for your consideration of our revised manuscript.

Sincerely,

Jie Lu

Associate Professor
School of Coal Engineering, Shanxi Datong University

School of mines, China university of Mining and Technology

Lujie0114@cumt.edu.cn
